# Supplementary material for: Spatial and Temporal Distribution of Bacterioplankton Molecular Ecological Networks in the Yuan River under Different Human Activity Intensity
Source: Microorganisms. 2021 Jul 19;9(7):1532. doi: 10.3390/microorganisms9071532 (PMC8306320; doi:10.3390/microorganisms9071532)
Supplement: Supplementary file 1 [file microorganisms-09-01532-s001.zip › microorganisms-1301785-supplementary.pdf]

## Supplementary information

**Table S1.** Location information and HAILS level (see Equ (1)) of land use patterns for the 16 sampling sites considered in the Yuan River basin.

| Region | Water sampling site | Farmland area percentage (%) | Forest area percentage (%) | Water area percentage (%) | Residential land area percentage (%) | Other area percentage (%) | HAILS (%) |
|---|---|---|---|---|---|---|---|
| Upper reaches | Y01 | 11.12 | 84.41 | 0.11 | 1.17 | 3.18 | 28.38 (16.58)[b] |
| | Y02 | 13.80 | 80.89 | 2.81 | 1.26 | 1.23 | |
| | Y03 | 40.18 | 45.52 | 0.87 | 11.83 | 1.6 | |
| | Y04 | 22.58 | 72.14 | 1.34 | 2.04 | 1.9 | |
| | Y05 | 34.95 | 58.95 | 0.79 | 2.96 | 2.36 | |
| Middle reaches | Y06 | 18.34 | 69.44 | 0.89 | 8.82 | 2.5 | 33.08 (16.79)[b] |
| | Y07 | 33.15 | 38.04 | 1.50 | 23.85 | 3.45 | |
| | Y08 | 32.80 | 60.26 | 1.55 | 4.05 | 1.34 | |
| | Y09 | 15.97 | 70.19 | 8.13 | 4.68 | 1.09 | |
| | Y10 | 10.00 | 78.85 | 8.94 | 1.23 | 0.98 | |
| | Y11 | 35.47 | 50.02 | 2.04 | 10.11 | 2.35 | |
| Lower reaches | Y12 | 35.18 | 44.69 | 2.03 | 15.90 | 2.2 | 66.09 (10.9)[a] |
| | Y13 | 44.67 | 33.43 | 2.72 | 16.50 | 2.68 | |
| | Y14 | 72.25 | 11.75 | 3.31 | 8.20 | 4.49 | |
| | Y15 | 63.75 | 21.26 | 3.80 | 6.51 | 4.69 | |
| | Y16 | 62.26 | 22.60 | 3.33 | 5.21 | 3.59 | |

* HAILS is the mean of five or six sampling sites with standard errors in parentheses. Within column different letters show statistical significance at ($p < 0.05$). One-way ANOVA was performed on three regions (n=3).

**Table S2.** Numbers of OTUs, sequence reads, relative percentage and taxonomic groups for bacterioplanktonic communities in the Yuan River.

| Taxa | OTUs | Sequences | | Phylum | Class | Order | Family | Genus | Species |
|---|---|---|---|---|---|---|---|---|---|
| | Richness | Reads abu. | Percentage | | | | | | |
| Wet season | 2074 | 892,792 | 48.89% | 41 | 90 | 173 | 314 | 607 | 1006 |
| Dry season | 3634 | 933,377 | 51.11% | 46 | 116 | 209 | 378 | 816 | 1556 |
| Whole | 3945 | 1,826,169 | 100.00% | 46 | 118 | 213 | 392 | 847 | 1619 |

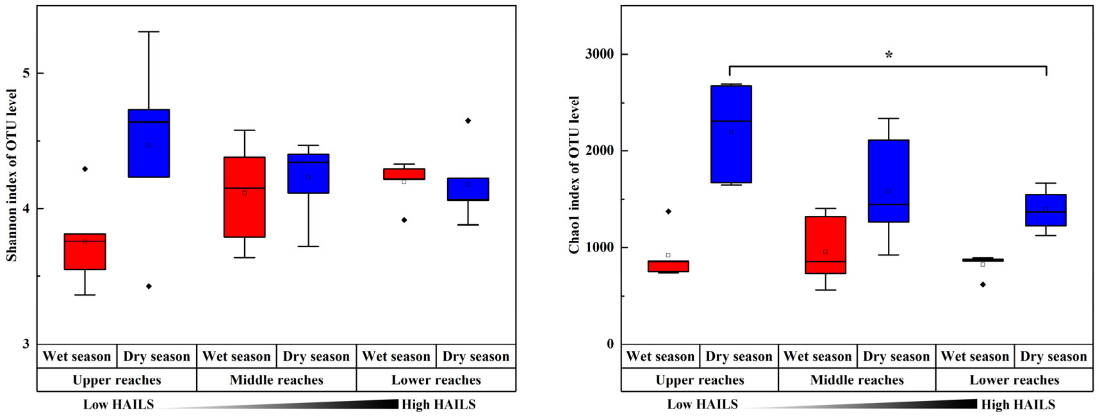


**Figure S1.** Comparison of Shannon diversity indices and Chao1 richness of communities from along the upper and downstream reaches of the Yuan River. Boxes show means ± SE, while whiskers show means ± SD. Wet season distributions are in red and dry season distributions in blue. The "*" indicates a statistically significant difference at $p < 0.05$ (one-way ANOVA).

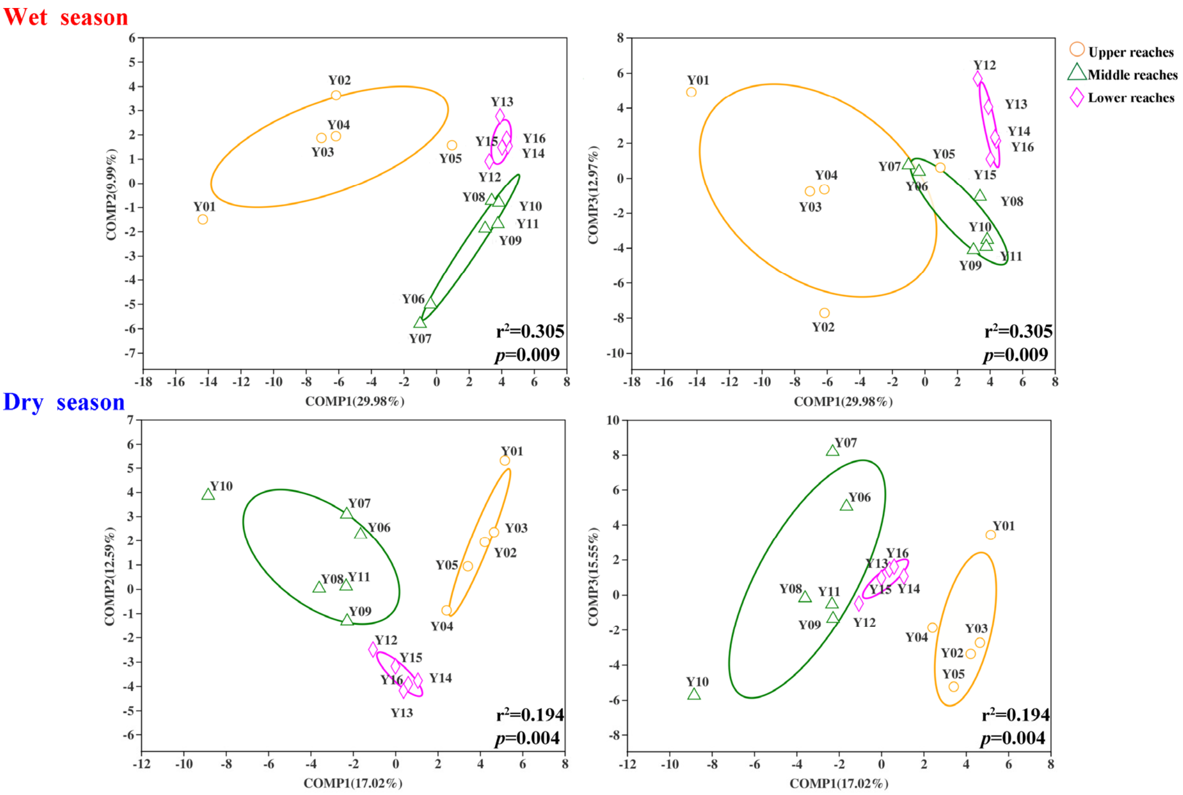


**Figure S2.** Partial Least Squares Discriminant Analysis score plots of bacterial communities.

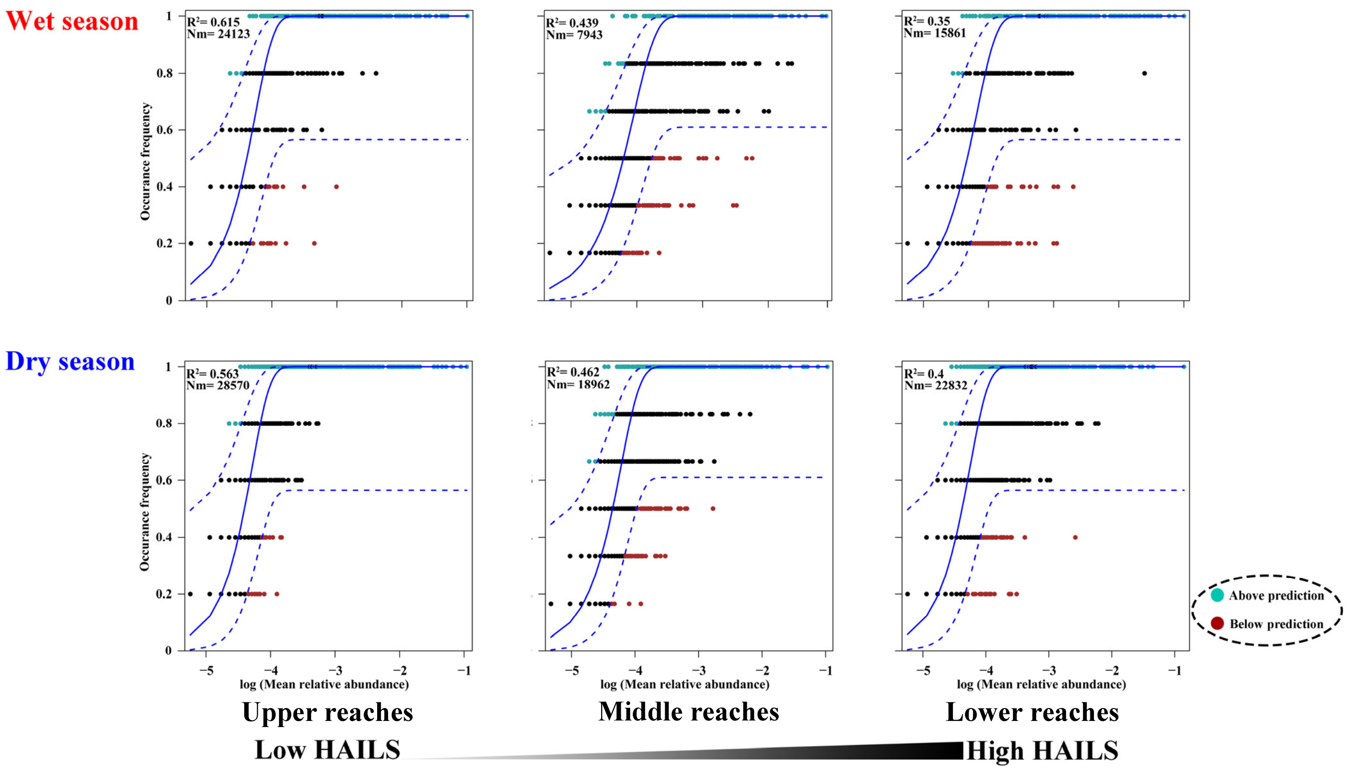


**Figure S3.** Fit of the neutral community model (NCM) of community assembly. The predicted occurrence frequencies for wet season, dry season, and all seasons, representing bacterioplanktonic communities in each respective time period. The solid blue lines indicate the best fit to the NCM as in Chen et al.[1], and the dashed blue lines represent 95% confidence intervals around the model prediction. OTUs that occur more or less frequently than predicted by the NCM are shown in different colors. Nm indicates the metacommunity size times immigration, $R^2$ indicates the fit to this model.

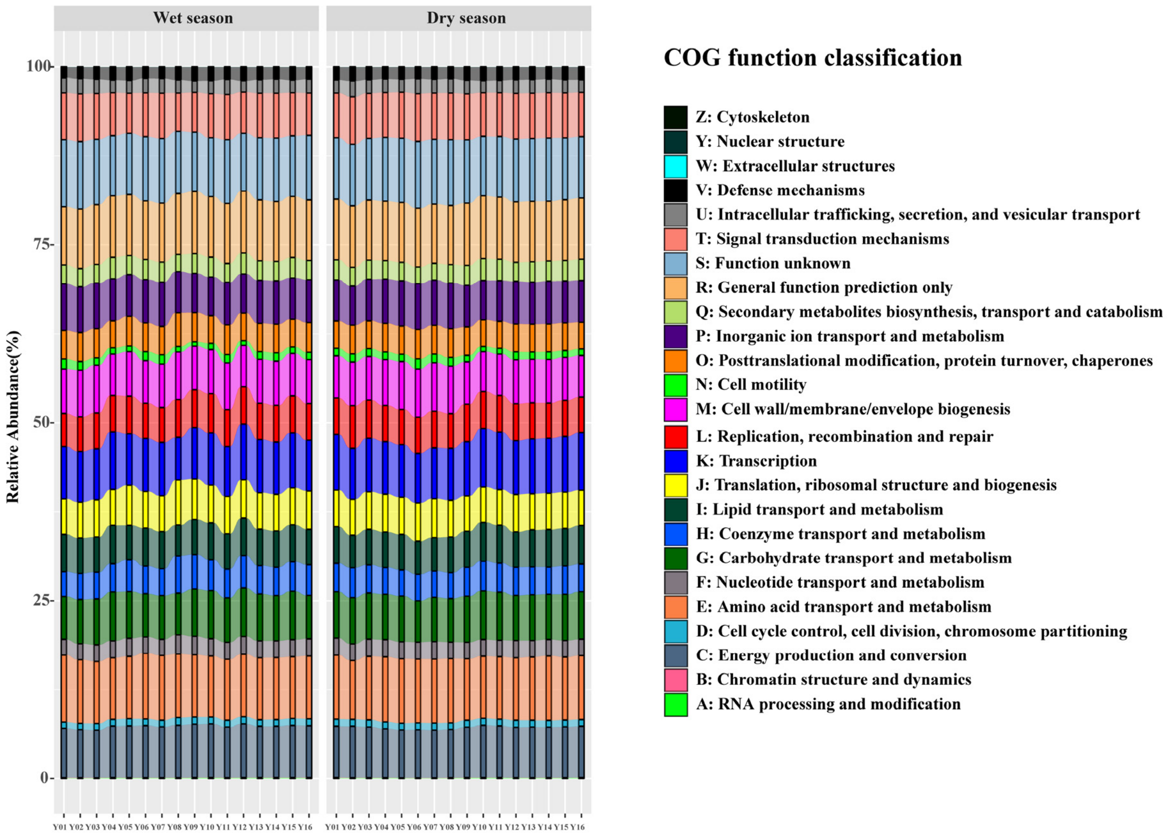


**Figure S4.** Relative abundance of PICRUSt inferred functions according to color shading (wet season and dry season). The relative abundances are calculated by averaging the abundances of bacteria.

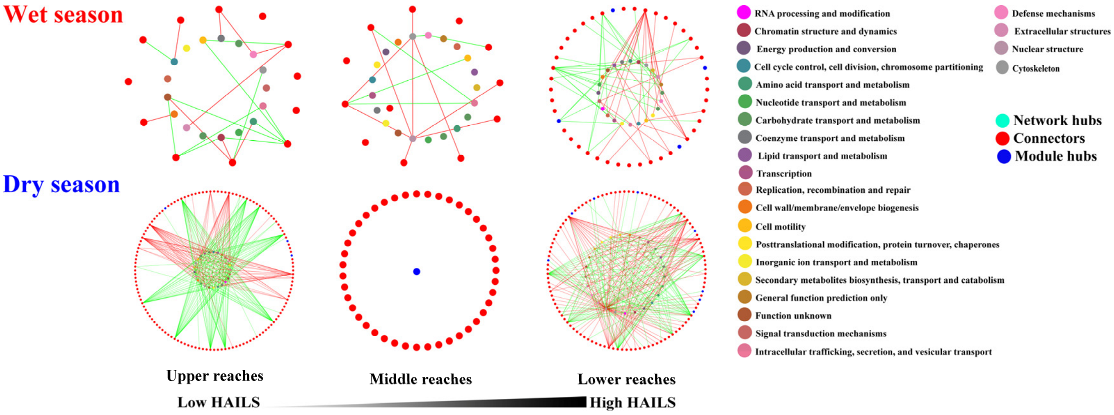


**Figure S5.** Mutualistic networks of interaction between keystone species community and functions during the wet and dry season in the Yuan River.

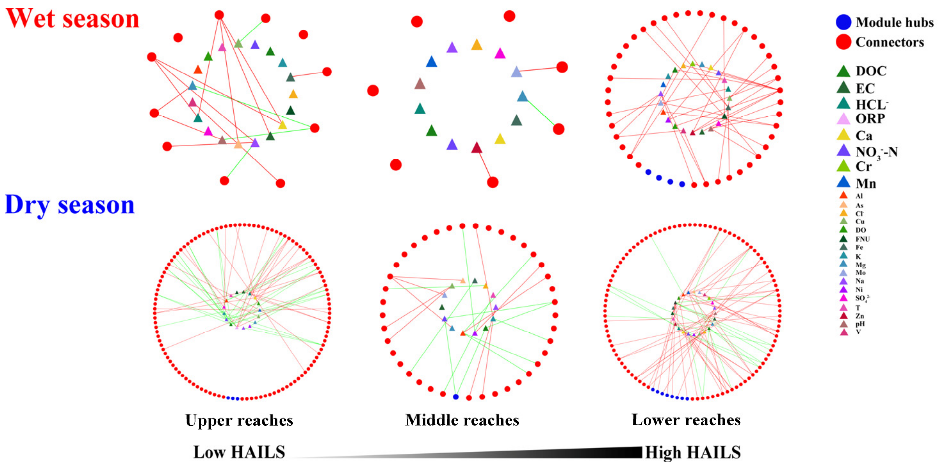


**Figure S6.** Mutualistic networks of interaction between keystone species community and water chemistry parameters during the wet and dry seasons in the Yuan River.

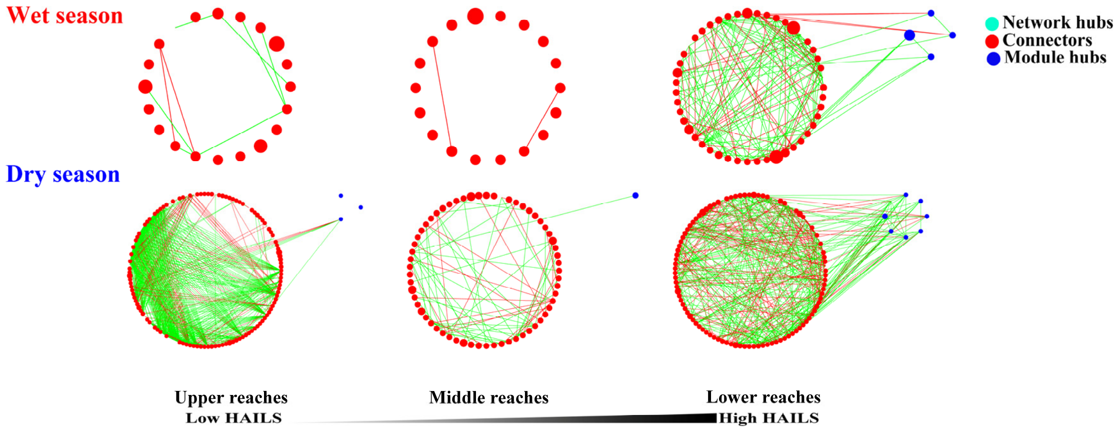


**Figure S7.** Mutualistic networks of interaction between keystone species community during the wet and dry seasons in the Yuan River.

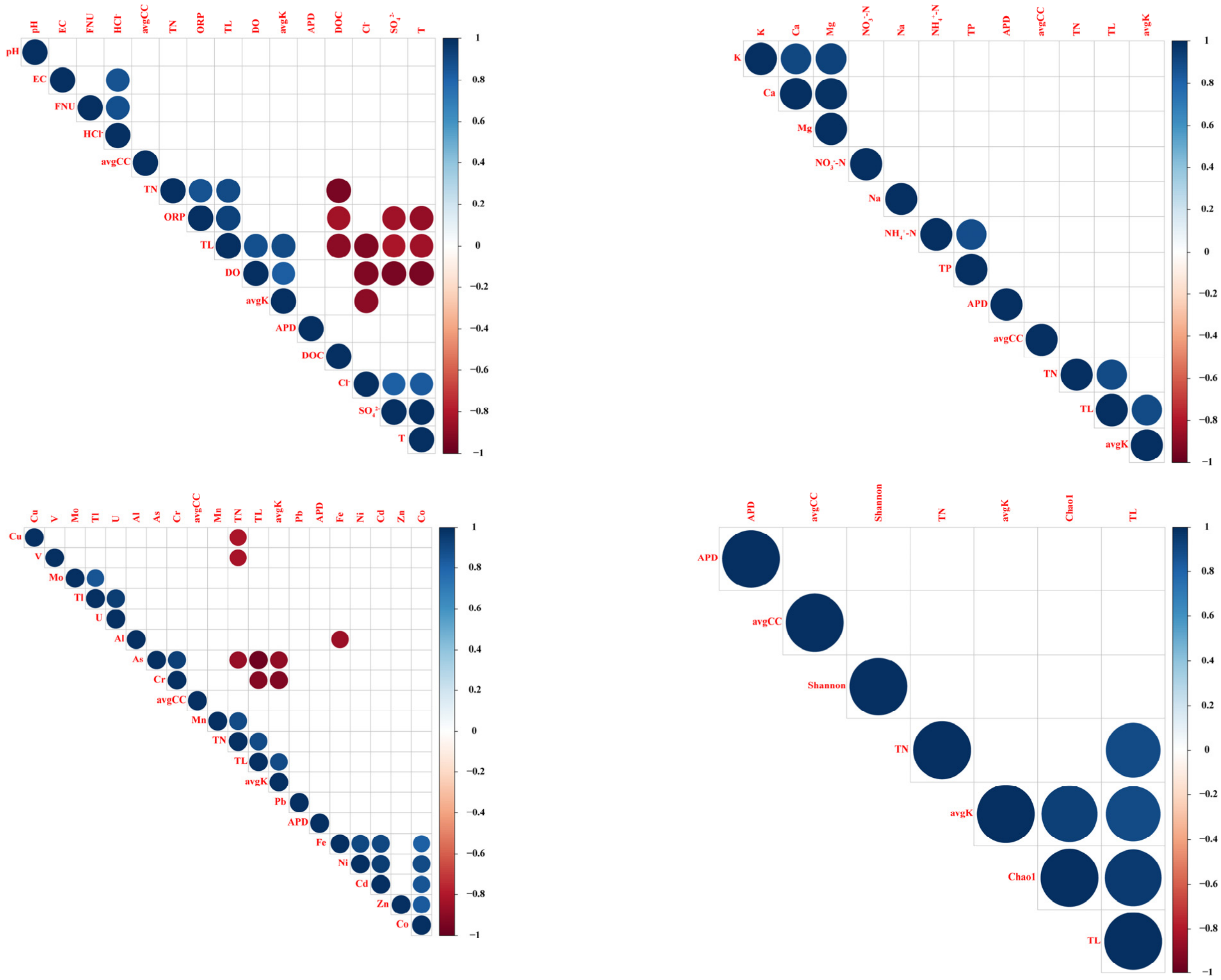


**Figure S8.** Pearson's correlation values between major modules network indexes (including avgK, TN, TL, avgCC and APD) with water chemistry parameter (including DO, ORP, $Cl^-$, $SO_4^{2-}$, T, DOC, pH, EC, FUN and $HCl^-$), nutrient variables (including K, Ca, Mg, $NO_3^-$-N, Na, $NH_4^+$-N and TP), heavy metals (including Pb, Fe, Ni, Cd, Zn, Co, Mn, Cu, V, Mo, Ti, U, As, Cr and Al) and diversity index (including Shannon index and Chao1 index), respectively

**Table S3.** Key topological features of the major modules in the bacterioplankton molecular ecological networks

| Network indexes | Wet season | | |
|---|---|---|---|
| | **Upper reaches** | **Middle reaches** | **Lower reaches** |
| Total nodes (TN) | 118 | 35 | 63 |
| Total links (TL) | 713 | 299 | 221 |
| Negative links (NL) | 256 | 103 | 52 |
| Positive links (PL) | 457 | 196 | 169 |
| Negative / Positive (NP) | 0.56 | 0.526 | 0.308 |
| Average degree (avgK) | 12.085 | 17.086 | 7.016 |
| Average clustering coefficient (avgCC) | 0.559 | 0.545 | 0.682 |
| Average path distance (APD) | 3.436 | 1.524 | 3.209 |

| Network indexes | Dry season | | |
|---|---|---|---|
| | **Upper reaches** | **Middle reaches** | **Lower reaches** |
| Total nodes (TN) | 275 | 120 | 182 |
| Total links (TL) | 7730 | 2062 | 1519 |
| Negative links (NL) | 1418 | 544 | 458 |
| Positive links (PL) | 6312 | 1518 | 1061 |
| Negative / Positive (NP) | 0.225 | 0.358 | 0.432 |
| Average degree (avgK) | 56.218 | 34.367 | 16.692 |
| Average clustering coefficient (avgCC) | 0.797 | 0.904 | 0.366 |
| Average path distance (APD) | 2.806 | 1.158 | 2.582 |

## References

1. Chen, W.; Ren, K.; Isabwe, A.; Chen, H.; Liu, M.; Yang, J. Stochastic processes shape microeukaryotic community assembly in a subtropical river across wet and dry seasons. *MICROBIOME* **2019**, *7*, 138.
